# The relationship between obesity and serum calcium level in a sample of Syrian people a cross sectional study

**DOI:** 10.1016/j.obpill.2025.100208

**Published:** 2025-09-02

**Authors:** Ola Faleh, Zaynab Alourfi

**Affiliations:** The National University Hospital, Division of Endocrinology, Damascus, Syria

## Abstract

**Introduction:**

Calcium is one of the most important elements in the human body playing a crucial role in various physiological functions. Its serum levels might be influenced by multiple factors. Obesity is a prevalent disease worldwide, and many studies have explored the relationship between obesity and serum calcium levels. This study aimed to investigate the association between Body Mass Index (BMI) and serum calcium.

**Material and method:**

This cross-sectional study conducted at the National University Hospital between 2023 and 2024 involved 206 participants. Serum calcium levels were measured. A fasting morning blood sample was collected from each subject, and all tests were performed at The National University Hospital.

**Result:**

The analysis showed no significant association between BMI and serum calcium levels, nor between age and serum calcium. However, after adjusting for age, the relationship between BMI and serum calcium strengthened and reached statistical significance, revealing an inverse correlation. This suggests that age acted as a partial confounding variable in the initial assessment.

**Conclusion:**

While the initial findings of this cross-sectional study initially revealed no significant relationship between body mass index (BMI) and serum calcium, after adjusting for age, an inverse relationship was found between BMI and serum calcium, with higher BMI associated with lower serum calcium levels.

## Highlights

• This cross-sectional study specifically examines the relationship between BMI and serum calcium levels.
• The findings indicate that there is no significant relationship between body mass index and serum calcium.
• After adjusting for age, the strength of the association increased and became statistically significant that there was an inverse relationship between BMI and body mass index indicating that age was a partial confounder.

## Graphical abstract

**Figure:**
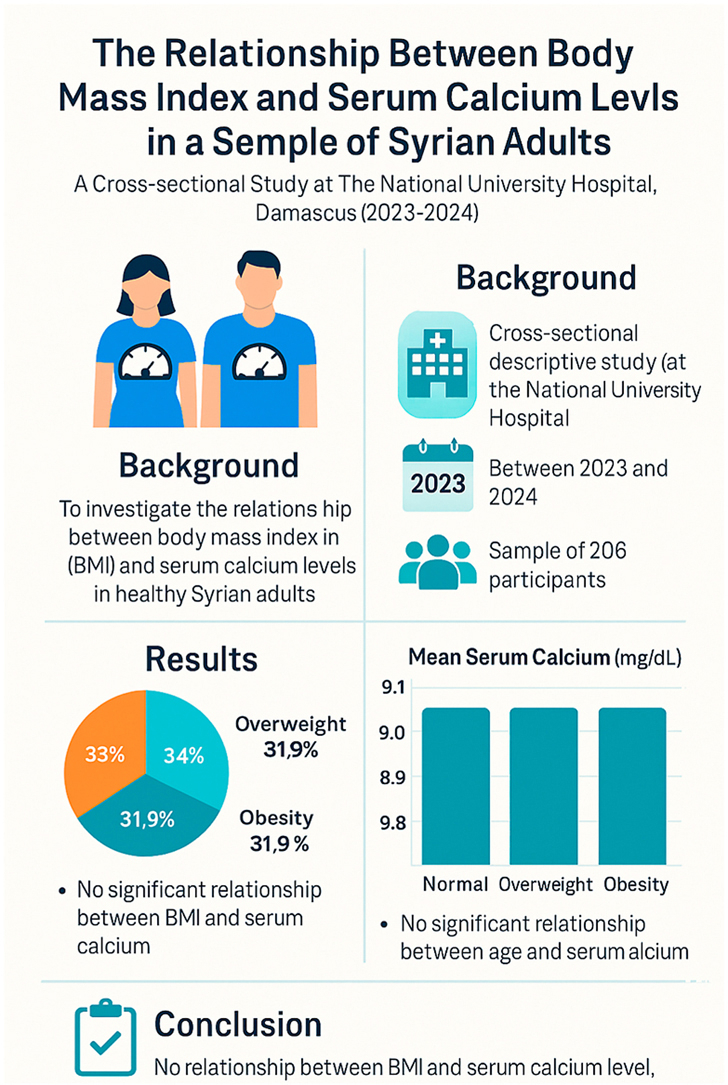

## 1 Introduction

Calcium (Ca2+) is one of the most significant elements in the human body, weighing approximately 1.5 kg [1]. More than 99 % of calcium incorporated into the mineral component of bone, while less than 1 % (about 20 mmol) is present in the extra cellular fluid (ECF) [2]. Hydroxyapatite, the phosphate form of calcium, constitutes the principal form of calcium in the human body, with over 98 % of this mineral being located in bones.

Calcium plays a crucial role in a wide range of physiological functions including muscle contraction as an increase in calcium levels within the cytoplasm of muscle cells facilitates this process. Additionally, calcium is vital for neurotransmitter release and synaptic plasticity, which are key components of nervous system function. It also influences hormone secretion and action, including insulin secretion from beta cells and the tissue responses to hormones such as glucagon and adrenaline [3]. Furthermore, calcium is important for blood coagulation by enhancing calcium levels inside platelets, which leads to their activation [4]. Lastly, it contributes to cell proliferation, differentiation, and survival [3].

Multiple factors might impact serum calcium levels, including age. As individuals age, calcium absorption from the intestine decreases due to a reduction in the TRPV6 channel and the intestines' resistance to 1,25-dihydroxyvitamin D. Additionally, there is often a decrease in 1,25-dihydroxyvitamin D production in the kidneys [5,6]. Gender also plays a role, as A study by Bosman, A. et al. found that calcium levels in females are generally higher than in males [7]. However, contrasting results were reported by Chen et al. [8], and Koek and colleagues suggested that women over the age of 45 have higher serum calcium levels than men, indicating age-dependent sex differences in serum calcium levels [9]. These discrepancies may be attributed to the influence of estrogen on calcium levels, Nordin et al. proposed that these sexual dimorphisms in calcium handling may result from changes in parathyroid hormone (PTH) levels and an increased sensitivity to PTH's effects on bone following menopause [10]. Factors such as smoking can also influence calcium levels, since smokers typically have lower vitamin D levels compared to non-smokers [11]. Diet can further affect these levels that inadequate dietary calcium intake is a global public health problem that disproportionately affects low- and middle-income countries (LMICs). However, measuring the calcium status of a population is challenging [12]. It is essential to note that the endocrine feedback system, including parathyroid hormone (PTH) and 1,25-dihydroxyvitamin D, strictly regulates serum calcium levels. Therefore, serum calcium cannot be reliably used as an indicator of total body calcium status or nutritional status [12,13]. The relationship between serum calcium levels and obesity remains a contentious topic. A study by Chen et al. found a positive correlation between waist circumference and serum calcium levels [8]. Similarly, Son et al. reported a positive relationship between serum calcium levels and body mass index (BMI) specifically in men [14]. Alrayah et al. also found that serum calcium is positively associated with BMI in both sexes [14]. Conversely, some studies have identified a negative relationship. Shahwan, Khattab, and Jairoun discovered a negative significant association between abdominal obesity and serum calcium levels among patients with diabetes [15]. Additionally, Mohieldein et al. found a negative correlation between serum calcium and obesity markers [16]. Overall, these conflicting findings underscore the complexity of the relationship between serum calcium and obesity.

Obesity is a disease that significantly affects human health. [17]. The World Health Organization (WHO), defines overweight and obesity as the accumulation of abnormal or excessive fat that is associated with increased health risk. Overweight is determined when body mass index >25 kg/m2 and obesity when BMI ≥30 kg/m2 [17]. BMI is not the most accurate metric of excess fat. it is the most commonly accepted way to determine the overweight or obesity, because it is easy to assess. According to the World Health Organization (, in 2022, 43 % of adults aged 18 and above were suffering overweight, and 16 % were suffering from obesity [18]. Having a high BMI is considered as a risk factor of various health problems, including metabolic disorders such as raised fasting glucose, impaired glucose tolerance, type 2 diabetes mellitus (T2DM), metabolic syndrome, hypertension, high triglycerides, low HDL-C, and fatty liver disease. Obesity is also associated with other well-known complications such as coronary heart disease, asthma, obstructive sleep apnea, orthopedic complications, and mental health issues such as depression and low self-esteem The prevalence of obesity is increasing globally, with acceleration in most regions of the world [17,19]. Studies have indicated that most individuals with obesity suffer from hidden hunger, which refers to a condition where individuals suffer from multiple micronutrient deficiencies despite having enough energy in their diet. This often results from consuming an energy-dense but nutrient-poor diet. It is estimated that more than two billion people around the world are affected, especially in low- and middle-income countries where people tend to rely on low-cost food staples and have limited dietary diversity [20,21] As a result, both adults and children with obesity tend to have lower levels of micronutrients compared to individuals with a normal weight [20,22]. AS was mentioned above, Calcium is an essential nutrient for the human body [1]. However, unlike other nutrients, the amount of calcium in the blood is affected by multiple factors apart from food intake [12]. Syria is experiencing difficult conditions that have resulted in a decline in overall health. This situation necessitates extensive research to better understand the health problems and challenges faced by the population. One of the most significant health issues in Syria is obesity, which is further exacerbated by the poor quality of food available. Thus, this study was conducted to explore the correlation between serum calcium levels and body mass index in a sample of Syrian people.

## 2 Material and methods

This cross-sectional non-intervention, non-randomized study was conducted at The National University Hospital in Damascus between 2023 and 2024. The study involved a sample of 207 adults who visited the hospital's outpatient department. The sample was calculated based on the following website: www.openepi.com/samplesizecalculator and according to the following standards:

The population size is the number of patients visiting the National University Hospital in Damascus, with a confidence level of 95 % and a confidence interval of 5 % based on this, the sample size is 206 as shown in Table (1) [13].

**Table (1):** Sample size calculation table.

	Kelsey	Fleiss	Fleiss with CC
Sample Size – Exposed	94	93	103
Sample Size-Nonexposed	94	93	103
Total sample size:	188	186	206

Sample Size: X-Sectional, Cohort, & Randomized Clinical Trials.

Two-sided significance level(1-alpha): 95.

Power (1-beta, % chance of detecting): 80.

Ratio of sample size, Unexposed/Exposed: nn1.

Percent of Unexposed with Outcome: 30.

Percent of Exposed with Outcome: 50.

Odds Ratio: 2.3.

Risk/Prevalence Ratio: 1.7.

Risk/Prevalence difference: 20.

The study protocol was approved by the Research Ethic Committee of Damascus Declaration of Helsinki and in line with STROCSS criteria [23]. the Research Ethic Committee of Damascus determined that this was an observational blood draw study without intervention, and thus did not require registration with ClinicalTrials.gov or an NCT number or its equivalent. The inclusion criteria for the study were healthy adults older than 18 years, while the exclusion criteria were pregnancy and lactation, established cardiovascular disease, hypertension or diabetes, endocrine disorders, cancers, consumption of dietary supplements, and taking medications that may affect serum calcium level The participants signed informed consent forms. They were instructed to fast overnight and provide a peripheral venous blood sample. The samples were analyzed for fasting plasma glucose, renal function test, serum calcium, phosphorus, albumin, and cell blood count. Serum calcium was measured by colorimetric assay using *o*-cresolphthalein complexone. The normal reference range for serum calcium was 8.4–10.4 mg/dl. The calcium level was corrected for albumin based on the following equation:

Corrected serum calcium = measured calcium(mg/dl) +0.8∗(4-albumin(mg/dl).

Information was taken about age, sex, and behavioral factors: physical activity by asking about walking which was categorized to less than 150 min/week, more than 150 min/week, or no walking, cigarette smoking (smoker or nonsmoker), as well as anthropometric factors (height, weight, BMI). Weight was measured using Seca scale (Itin Scale Co., Inc. Germany) with precision to the nearest 100 g Weight (in kilograms) while subjects had light clothes and no shoes. Height was measured using a calibrated measuring board manufactured by Seca with precision to the nearest 0.1 cm. BMI was calculated by dividing weight in kilograms by height in meters squared. Blood pressure was taken in the right upper arm while the participant is in a sitting position and using an aneroid manometer after a 5-min rest.

The statistical study was conducted by using the SPSS statistical software.

## 3 Results

### 3.1 General characteristics of subjects

A total of 207 apparently healthy participants 185 (89 %)of them were female, and 21(10.2 %) of them were men); 207 blood samples were drawn. The mean (SD) age of the participants was 43.3 years, ranging from 18 to 74 years. 33 % of participants had normal weight. 34 % of participant had overweight, and 31.9 % of participant had obesity. The mean corrected serum calcium was 9.1 mg/dl ranging from 6.3 to 10.9 mg/dl. The general characteristics o subject was shown in Table (2), Table (3).

**Table (2):** General characteristic.

Variables	Normal ranges	N	Mean	Median	Mode	Std. Deviation	Range	Min	Max
**Age (years)**	–	207	43.33	44	40	12.4	56	18	74
**Height(cm)**	–	207	161.32	160	155^a^	7.22	42	143	185
**Weight (kg)**	–	207	73.73	72	71	16.45	105	46	151
**Body mass index (BMI) (kg/m^2)^**	–	207	28.3	27.53	20.81	5.9	39.25	18.29	57.54
**Systolic blood pressure (mmHg)**	9–14	196	11	12	12	1.016	5	9	14
**Diastolic blood pressure (mmHg)**	6–8	197	7	7	7	0.87	4	5	9
**Calcium (mg/dl)**	8.4–10.4	207	9.29	9.3	9.6	0.54	3.6	7.2	10.8
**Blood glucose (mg/dl)**	60–99	189	90.3	89	88	9.19	58	65	123

**Table (3):** General characteristic.

		N	%
**Physical activity**	**No walking**	72	47.4
	**Less than 150/min/week**	31	20.4
	**More than 150/min/week**	49	32.2
	**total**	**152**	**100**

	**N**	**%**
smoker	**21**	**11.7**
nonsmoker	**159**	**88.3**
total	**180**	**100**

### 3.2 The relationship between BMI and corrected serum calcium

**Figure (1):**
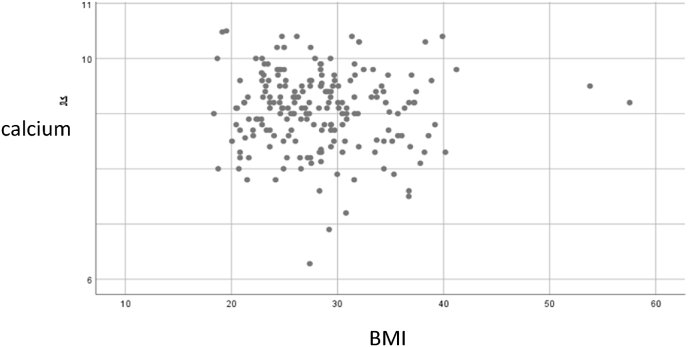
The relationship between body serum calcium and body mass index (BMI).

The participants were divided into three BMI categories: individuals with normal weight, individuals with overweight, and individual with obesity. Differences in corrected serum calcium levels among these groups were assessed using a one-way ANOVA test. The results are presented in Table (4) and figure (1). No statistically significant differences were observed between the groups.

**Table (4):** The correlation between body mass index (BMI) and serum calcium the correlation was significant at (p−value≤0.05).

BMI Category	Sample Size (n)	Mean	Std. Deviation	95 % CI for Mean (Lower–Upper)	Min	Max	F	p	Effect Size (η^2^)
Normal weight BMI(18.5–24.9)kg/m^2^	69	9.3	0.7	9.1–9.4	7.8	10.9			
Overweight BMI(25–29.9)kg/m^2^	72	9.0	0.7	8.8–9.2	6.3	10.4	2.311	0.102	0.022
Obesity BM ≥ I30k/m^2^	66	9.1	0.8	8.9–9.3	7.2	10.7			
Total	207	9.1	0.8	9.0–9.2	6.3	10.9

Notes.

• Group labels have been clarified to match standard terminology.
• Sample sizes (n) are displayed next to each group.
• Effect size (η^2^) was calculated from the ANOVA F-value and degrees of freedom.
• η^2^ = 0.022 indicates a very small effect size, meaning BMI explains about 2.2 % of the variance in corrected serum calcium levels.

### 3.3 The correlation between BMI and age, and serum calcium

The correlation between corrected serum calcium, and BMI and age was studied by using Pearson Correlation, and Pearson correlation value the result is shown in Table (5). There was no correlation between corrected serum calcium, and BMI and age.

**Table (5):** The correlation between calcium, and body mass index (BMI) and age.

Variables	Pearson Correlation		
	N	Pearson Correlation value	p.value
**Corrected serum calcium**	207	−0.051	0.468
**BMI**	207		
**Corrected serum calcium**	207	0.087	0.216
**Age**	207		

∗the correlation was significant at (p−value≤0.05)

### 3.4 Statistical relationship between corrected serum calcium values and body mass index (BMI) after controlling for confounding factors (age, sex)

The relationship between corrected calcium levels∗ (continuous quantitative variable) and BMI category (binary: normal vs. with overweight/obesity) was examined using linear regression.

The models were built in stages to assess the effect of confounding factorsf ∗Statistically significant at p < 0.05.

Model 1: Unadjusted (BMI only)
Model 2: Adjusted for age
Model 3: Adjusted for age and sex

Regression coefficients (B), 95 % confidence intervals, p-values, as well as the coefficient of determination (R^2) and its change (Delta R^2) were reported to estimate the variation explained by adding variables. Table (6) shows the results of the different models according to the inclusion of confounding factors Linear regression analysis of the relationship between corrected calcium and BMI category (with overweight/obesity vs. with normal weight) adjusted for age and sex Model Variables Included B 95 % CI for BP-value R^2 Delta R^2.

**Table (6):** Relationship between serum calcium values and body mass index (BMI) after controlling for confounding factors (age, sex).

model	Variables included	B	95 % CI for B	P-value	R^2^	Δ R^2^
**1**	**BMI**	−0.217	−0.436, 0.002	0.053	0.018	0.018
**2**	**BMI**	−0.233	−0.452, −0.013	0.038∗	0.029	0.010
	**age**	0.006	−0.002, 0.014	0.148		
**3**	**BMI**	−0.233	−0.453, −0.013	0.038∗	0.029	0.000
	**age**	0.006	−0.002, 0.015	0.149		
	**sex**	0.006	−0.334, 0.346	0.972		

BMI and calcium in this sample.

Note: BMI - (0: normal, reference category); 1: overweight/obese.

Stage 1 (BMI only): B = −0.217, indicating that individuals in the overweight/obese category had an average corrected calcium level lower by 0.217 compared to the normal BMI category.

P-value = 0.053, which is close to significance but not statistically significant at the 0.05 level.

R^2 = 0.018, meaning the model explains only 1.8 % of the variance in calcium levels - very little.

Interpretation: There is an indication of an inverse relationship between BMI and calcium, but it is weak and not statistically significant.

Stage 2 (BMI + Age):

B = −0.233, showing that the effect size increased slightly after adjusting for age (from −0.217 to-0.233).

∗Corrected serum calcium = measured calcium(mg/dl) +0.8∗(4-albumin(mg/dl)

P-value = 0.038, meaning the relationship became statistically significant after adjusting for age.

Delta R^2 = 0.010, indicating that age added an additional 1 % of explained variance.

Interpretation: Age appears to be a confounding factor; including it in the model made the.

BMI-calcium relationship clearer and statistically significant.

Stage 3 (BMI + Age + Sex):

B and P-value remained almost identical to Stage 2 (P = 0.038, B = −0.233).

Delta R^2 = 0.000, indicating that sex did not add any additional explained variance.

Interpretation: Sex is neither a confounding nor an influential factor in the relationship between.

## 4 Discussion

The sample had a bias toward females and older individuals, with women making up 89 % of the participants and the average age being 43.3 years. This distribution reflects the demographics of visitors to the National University of Damascus, where there is a higher proportion of women and older attendees.

This study found no significant correlation between body mass index (BMI) and serum calcium levels. This conclusion was reached using One-Way ANOVA and Pearson Correlation analysis. These findings are consistent with research conducted by Jumaahm, M.K., A.H.A. Alhamza, and A.A. Mansour in Iraq [24]. Since Iraq is geographically close to Syria and shares common ethnicity, traditions, and lifestyles, this similarity may help explain the comparable results. Many factors play roles in calcium hemostasis. The most important are PTH and 25OH vitamin D, which regulate Serum and extracellular calcium concentrations in mammals within a narrow physiologic range because maintaining calcium levels is optimal for many cellular functions [4], which could explain the absence of significant correlation between body mass index and serum calcium. When blood calcium levels decrease due to inadequate dietary intake, PTH initiates several responses: 1. It increases calcium reabsorption in the kidneys, specifically from the ascending loop of Henle, the distal convoluted tubule, and the collecting duct. 2. It converts 25-hydroxyvitamin D into its active form, 1,25-dihydroxyvitamin D. 3. It stimulates bone resorption by activating osteoclasts, which release calcium from bones into the bloodstream [25]. The active form of vitamin D, 1,25-dihydroxyvitamin D, also plays a crucial role by:

1. Enhancing bone resorption through the continued activation of osteoclasts. 2. Increasing calcium absorption from the intestines. This is achieved by promoting passive intercellular absorption and inhibiting certain binding proteins such as Claudin 2, Claudin 12, and Cadherin 17 [26]. So, it is important to note that measuring blood calcium levels alone is insufficient to assess calcium stores in the body or dietary intake [12,13,26]. Additionally, calcium within cells is managed by various organelles that function as calcium storage sites the most important of which are: Endoplasmic Reticulum, Mitochondria, and Lysosomes. These organelles contain a calcium concentration approximately 10,000 times higher than its concentration in the cytoplasm. When the cell transitions from a resting state to an active state to perform its functions, calcium levels in the cytoplasm rise as a result of its release from these storage sites [27]. Other factors that influence calcium hemostasis include age, gender, and diet. The analysis showed no significant association between BMI and serum calcium levels, nor between age and serum calcium. However, after adjusting for age, the relationship between BMI and serum calcium strengthened and reached statistical significance, revealing an inverse correlation. This suggests that age acted as a partial confounding variable in the initial assessment.” When sex was included in the model, there was no significant change in effect size or significance, and sex did not account for any additional variance in calcium levels (Delta R^2 = 0.000), suggesting that it did not play a confounding role in this sample. Overall, these results imply that a higher BMI may be linked to lower corrected calcium levels, with this association becoming clearer after controlling for age. These results may indicate the hidden hunger affecting many Syrians, highlighting the need to evaluate the nutritional status of the Syrian population, particularly among those with obesity and the elderly Similarly, others have suggested a negative correlation due to low vitamin D levels in obese individuals [15,16,28], which was not measured in this study and needs to be evaluated in addition to another hormone like PTH, in the Syrian population.

Other studies have shown conflicting results, with some indicating a positive correlation between serum calcium and body mass index due to elevated levels of PTH and inflammatory cytokines. Furthermore, the Pearson correlation analysis revealed no association between age and serum calcium levels. This finding aligns with a study conducted by Jafari-Giv et al. in Iran [28], a country closes to Syria that shares similar traditions and ethnic backgrounds. Some research has indicated that variations in calcium levels with age can differ across various populations and sub-populations [29]. Moreover, the lack of significant correlation between age and serum calcium may be attributed to the relatively narrow range of participants, the majority of whom were older adults. These findings contrast with those of study of Son, S.-W. et al., conducted in Korea, which demonstrated a decline in calcium levels with advancing age [30]. Some studies have reported that intestinal calcium absorption decreases with age due to a reduction in the TRPV5 channel, as well as the development of resistance to 1.25- dihydroxy D at the intestinal level and diminished synthesis of 1,25-dihydroxyvitamin D in the intestine [5,6]. The strength of this study is its location at The National University Hospital, the central medical center of Syria, where people from all over the country come to receive care.

### 4.1 Limitation

Our study has several limitations. First, as a cross-sectional study, it cannot establish cause-and-effect relationships. Second, we did not consider variations in calcium intake between individuals with obesity and those without. Additionally, we did not measure hormones that influence calcium levels, such as parathyroid hormone (PTH) and vitamin D.

## 5 Conclusion

This cross-sectional study was conducted at the National University Hospital and included 207 participants to examine the relationship between serum calcium levels and obesity. While the initial findings of this cross-sectional study initially revealed no significant relationship between body mass index (BMI) and serum calcium, after adjusting for age, an inverse relationship was found between BMI and serum calcium, with higher BMI associated with lower serum calcium levels.

### 5.1 Takeaway messages

o The initial findings of this cross-sectional study initially revealed no significant relationship between body mass index (BMI) and serum calcium
o Adjusting for age, an inverse relationship was found between BMI and serum calcium, with higher BMI associated with lower serum calcium levels.

These findings may help bring context to the assessment of calcium levels in patients with overweight and obesity.

## Ethics approval and consent to participate

The study was approved by the Biomedical Research and Ethics Committee of the University of Damascus, the BMREC Ethics Committee (protocol ID MD-15025-438), Faculty of Medicine.

## Availability of data and material

All relevant data are presented in the main paper.

## Authors' contributions

The study was conceptualized, designed, and conducted by Ola Faleh. Data collection, analysis, and interpretation were performed by Ola Faleh, who also drafted the initial manuscript. Zeinab Alourfi critically reviewed the manuscript for intellectual content, provided editorial suggestions, and approved the final version for submission.

## Declaration of artificial intelligence (AI) and AI-assisted technologies utilized in the writing process

None.

## Funding

None.

## Declaration of competing interest

The authors declare that they have no known competing financial interests or personal relationships that could have appeared to influence the work reported in this paper.
